# Mechanisms underlying acupuncture therapy in chronic kidney disease: A narrative overview of preclinical studies and clinical trials

**DOI:** 10.3389/fneph.2022.1006506

**Published:** 2022-11-09

**Authors:** Xinyin Liu, Xiaoran Wang, Hongzhen Ma, Wen Zhang

**Affiliations:** ^1^ The First Clinical Medical College, Zhejiang Chinese Medical University, Hangzhou, China; ^2^ Department of Nephrology, The First People’s Hospital of Hangzhou Lin’An District, Hangzhou, China; ^3^ Department of Nephrology, The First Affiliated Hospital of Zhejiang Chinese Medical University (Zhejiang Provincial Hospital of Chinese Medicine), Hangzhou, China

**Keywords:** complementary therapy, acupuncture, therapeutic mechanism, chronic kidney disease, end-stage renal disease

## Abstract

Chronic kidney disease (CKD) is associated with high incidence, low awareness, and high disability rates among the population. Moreover, the disease significantly affects the physical and mental health of patients. Approximately 25% of patients with CKD develop end-stage renal disease (ESRD) within 20 years of diagnosis and have to rely on renal replacement therapy, which is associated with high mortality, heavy economic burden, and symptoms including fatigue, pain, insomnia, uremia pruritus, and restless leg syndrome. Currently, the means to delay the progress of CKD are insufficient; therefore, developing strategies for delaying CKD progression has important practical implications. In recent years, more and more people are accepting the traditional Chinese medical technique “acupuncture.” Acupuncture has been shown to improve the uncomfortable symptoms of various diseases through stimulation (needling, medicinal moxibustion, infrared radiation, and acupressure) of acupoints. Its application has been known for thousands of years, and its safety and efficacy have been verified. As a convenient and inexpensive complementary therapy for CKD, acupuncture has recently been gaining interest among clinicians and scientists. Nevertheless, although clinical trials and meta-analysis findings have demonstrated the efficacy of acupuncture in reducing albuminuria, improving glomerular filtration rate, relieving symptoms, and improving the quality of life of patients with CKD, the underlying mechanisms involved are still not completely understood. Few studies explored the correlation between acupuncture and renal pathological diagnosis. The aim of this study was to conduct a literature review summarizing the currently known mechanisms by which acupuncture could delay the progress of CKD and improve symptoms in patients with ESRD. This review help provide a theoretical basis for further research regarding the influence of acupuncture on renal pathology in patients with CKD, as well as the differences between specific therapeutic mechanisms of acupuncture in different renal pathological diagnosis. The evidence in this review indicates that acupuncture may produce marked effects on blocking and reversing the critical risk factors of CKD progression (e.g., hyperglycemia, hypertension, hyperlipidemia, obesity, aging, and anemia) to improve the survival of patients with CKD *via* mechanisms including oxidative stress inhibition, reducing inflammatory effects, improving hemodynamics, maintaining podocyte structure, and increasing energy metabolism.

## 1 Introduction

Chronic kidney disease (CKD) refers to abnormal renal structure or function caused by various reasons or an unexplained decrease in the glomerular filtration rate (GFR<60 mL/min) for >3 months (1). In 2012, the *Lancet* published the first nationwide cross-sectional survey of CKD in China, showing that there were approximately 120 million patients with CKD, which had a 10.8% prevalence rate among adults (2). According to the five insights of the 2019 Global Disease Burden Study, exposure rate to the risk factors of injury and disability caused by CKD has also increased, second only to hypertension, hyperglycemia, obesity, environmental pollution, and social factors (3). Furthermore, approximately 25% of patients with CKD develop end-stage renal disease (ESRD) within 20 years of diagnosis (4). Patients with ESRD have a high mortality rate and require long-term maintenance dialysis, which incurs substantial medical costs and places a heavy economic burden on families, society, and the country. Currently, the etiology and pathogenesis of CKD are unclear, and there is a lack of effective treatments.

### 1.1 Treatment of CKD in Western medicine

The main treatments of CKD target preventing ESRD to reduce mortality. Modern medicine has made several attempts to treat this disease, including dealing with its complications (e.g., renal hypertension, hyperlipidemia, proteinuria) using glucocorticoids, cytotoxic drugs, immunosuppressants, and biological agents based on individual patient characteristics and renal pathology (5). However, there are currently no ideal treatment schemes, and this disease has become a significant public health concern worldwide. Hence, it is necessary to explore novel methods for its prevention and treatment.

### 1.2 Associated symptoms and symptomatic treatment

The considerable symptomatic burden associated with CKD greatly affects the quality of life of patients. Common symptoms include fatigue, pain, sleep disorders, restless leg syndrome (RLS), and chronic pruritus, although with considerable variations related to symptom definition, period of prevalence, and levels of severity (6–8). The first-line intervention of pain and pruritus is mainly medication, but their use and management have limitations, and nonpharmacologic approaches have therefore attracted attention. For example, replacement therapy has been shown to be feasible and effective against fatigue and sleep disorders (9).

### 1.3 Acupuncture and CKD

Acupuncture is an essential component of traditional Chinese medicine (TCM) and substitute auxiliary therapies. According to the TCM theory, there are 12 main and collateral channels on the human body surface and 361 classical acupuncture points on these channels (10). Acupuncture can adjust the qi and blood of the meridians and viscera, improve various uncomfortable symptoms, and treat diseases by stimulating different acupoints. For the purpose of this review, acupuncture is considered as a generalized concept, including procedures involving insertion of fine needles into the skin or deeper tissues at specific locations (acupoints) of the body which are then manipulated manually, electrically, or with combined moxibustion; pressure on the acupoints with fingers; or application of infrared radiation on acupoints instead of fine needles.

#### 1.3.1 Effectiveness

Acupuncture has been proven to reduce urine protein levels and improve estimated GFR (11–18). Meanwhile, some studies also focused on the effects of acupuncture on hemodynamics (19) and renal interstitial fibrosis (20–24). Factors such as hyperglycemia, hypertension, hyperlipidemia, obesity, pain, aging, and anemia have a profound relationship with CKD progression (25). Considering that acupuncture can be effective in improving these aspects (26–30), it is believed that acupuncture may improve the prognosis of patients with CKD by controlling the above risk factors. In addition, acupuncture has the potential to alleviate various ESRD-related symptoms (e.g., pain, uremic pruritus [UP], RLS, and sleep disorders) (31–33). The therapeutic effect of acupuncture on CKD cannot be explained entirely by the bidirectional regulation of nerves, which is generally considered the main effective mechanism (34). However, a number of large-scale, randomized controlled clinical trials are still needed to clarify the indications of acupuncture before it can be used widely in clinical practice, especially as the mechanisms by which acupuncture affects disease mechanisms can be quite complex.

#### 1.3.2 Security

The safety of acupuncture has been widely confirmed in clinical practice. A prospective observational study on acupuncture for chronic pain in Germany included 454920 patients, of whom more than 30% were over 60 years old, and reported mild side-effects (pain, hematoma, and bleeding) in 7.9% of patients. Only 13 patients suffered serious adverse events, including pneumothorax, hypertension, hypotension, asthma attacks, and aggravation of suicidal thoughts (35). The safety of acupuncture for CKD has also been proved. A systematic review of 55 randomized controlled trials showed that the most common side-effects associated with needling therapy and acupressure therapy were elbow soreness and bleeding and intradialytic hypotension and dizziness, respectively, and that no adverse effects were reported for moxibustion therapy (36). Some researchers believe that some side-effects of acupuncture are due to malpractice by acupuncturists, which can be avoided by strengthening training (37). The World Health Organization recommends at least 1568 hours of training to meet the basic requirements of acupuncture practitioners of ensuring clinical efficacy and patient safety (38).

However, although acupuncture is one of the safest replacement therapies, especially when provided by well-trained acupuncturists, to the best of our knowledge, the recent progress related to the use of acupuncture in treating CKD has not been summarized. This review aimed to assess the beneficial effects and current known mechanisms of acupuncture with regard to CKD and ESRD-related symptoms. We believe that acupuncture may have a significant impact on the associated risk factors for blocking or reversing the progress of CKD and alleviating the discomfort of patients, thus improving their prognosis.

## 2 Methods

We designed our literature review to include basic and clinical studies that addressed the effects and mechanisms underlying the effects of acupuncture treatment in CKD. The PubMed databases were queried for full-text studies published between January 1, 2000 and August 31, 2022 in English or Chinese using the following keywords: “acupuncture” and “kidney”.

Inclusion criteria:

1. Description of specific mechanisms of how the acupuncture treatment exerted its effects
2. Exclusive use of acupuncture to treat CKD and related symptoms
3. Related studies cited in these articles

Initially, 747 published articles were identified, of which only 46 articles matched the inclusion criteria and were reviewed. We excluded one withdrawn article, two articles unable to find partial results of changes in renal function, and two articles with imprecise test design that does not control variables. The references of the remaining 41 articles identified an additional 6 articles that also matched our inclusion criteria, which were also included in the final review, resulting in 47 articles in total (**Table 1**). The studies included randomized controlled animal experiments and clinical experiments and involved kidney injury. Additionally, we conducted a supplementary literature search on hyperlipidemia, which was not identified in the previous search but has been proved to be an important independent risk factor for the development of CKD.

**Table 1:** Summary of studies in chronic renal injury, and renal physiological function and ESRD-related symptoms (2000-2022).

Ref.	Type of study	Species	Sample size	Related disease/symptom	Acupuncture therapy	Result	Conclusion	Mechanism
(12) Yu et al.	RCT	Human	59	CKD	Needling at Hegu (LI4), EA at “Zusanli” (ST36) and “Taixi” (KI3) for 20 minutes, once per week, for 12 weeks	Reduced Scr and increased estimated GFR level	Improved renal function	None
(15) Zhu et al.	RCT	Human	106	CKD	Needling at “Shenguan”, “Dihuang”, and “Renhuang” for 30 minutes, MO at these acupoints for 20 minutes, once per day, the interval of 2 days once every 10 treatments, for 2 months	Reduced 24h-UP and red blood cell count of urinary sediment, and increased creatinine clearance rate	Improved renal function	None
(16) Paterno et al.	Preclinical study	Male Wistar rats	21	CKD	EA at “Zusanli” (ST36) and “Taixi” (KI3) for 20 minutes, EA and MO at “Shenshu” (BL23) for 2 minutes, twice a week, for 8 weeks	Improved urine volume, Scr, decreased 24h-UP, blood pressure, glomerulosclerosis and tubulointerstitial fibrosis indices	Attenuated the progression of renal disease	None
(17) Nie et al.	RCT	Human	180	Chronic allograft nephropathy	Needling at “Sanyinjiao” (SP6), “Diji” (SP8), “Yinlingquan” (SP9), “Xuehai” (SP10), etc. in spleen-meridian group; “Taixi” (KI3), “Zhaohai” (KI6), “Fuliu” (KI7), “Ciliao” (BL32), etc. in kidney-meridian group, for 30 minutes, once per day, the interval of 2 to 3 days once every 10 treatments, for 20 treatments	Reduced 24h-UP in all groups, and decreased Scr in spleen-meridian group	Relieved the damage of transplant kidney	None
(18) Mao et al.	RCT	Human	60	Idiopathic membranous nephropathy	MO at “Shenshu” (BL23), “Pishu” (BL20), “Guanyuan” (CV4), “Zusanli” (ST 36) and “Sanyinjiao” (SP6), for 30 minutes, once a day, 5 days a week, the interval of 2 days once every 5 treatments, for 6 months	Decreased the total TCM syndrome scores, the levels of 24h-UP, the blood coagulation indexes, TC and TG, increased the levels of ALB	Improved the clinical symptoms, renal function and renal microcirculation	None
(19) Matsumoto-Miyazaki et al.	RCT	Human	43	CKD	MO at “Shenshu” (BL23), for 4 minutes, 3 times in succession	Reduced resistive index	Decreased renal vascular resistance	None
(21) Li et al.	Preclinical study	Male SD rats	51	FSGS	MO at “Shenshu” (BL23) in one group and “Geshu” (BL17) in another group, for 30 minutes, every other day, for 12 weeks	Reduced UP, Scr, urea nitrogen, and serum uric acid, decreased renal α-SMA, fibronectin and TGF-β, increased podocin protein, nephrin protein and mRNA	Alleviated podocyte injury and inhibits RIF, improved renal function	Increase renal podocin and nephrin protein expressions, maintain the structural integrity of podocyte septa. Regulate renal tubular epithelial-mesenchymal transition and ECM integrity
(22) Zuo et al.	Preclinical study	Male adult New Zealand white rabbits	30	CRF	Needling at “Shenshu” (BL23), “Mingmen” (DU4) and “Pishu” (BL20), for 30 minutes, once a day, for 36 days	Reduced the levels of TNF−α, Smad3, ILK and TGF−β expression, decreased the concentrations of TGF−β, IL−8, TNF−α and IL−1β in blood serum, and increased eNOS expression	Relieved RIF, promoted the recovery of renal function	Regulate TGF-β-related pathways, decreased the levels of inflammation-associated cytokines, and attenuated RIF *via* the TGF-β/Smad pathway
(23) Zhang et al.	Preclinical study	Male SD rats	20	CRF	EA at “Sanyinjiao” (SP 6), “Taixi” (KI3) and “Shenshu” (BL23) for 20 minutes, once daily, for 30 days	Decreased the body weight, Scr and BUN levels and the expression of beta-catenin in the renal tissue	Relieved RIF, improved the renal function	Reduce the expression of beta-catenin in the renal tissue
(24) Li et al.	Preclinical study	Male SD rats	36	FSGS	EA at “Sanyinjiao” (SP 6), “Taixi” (KI3) and “Shenshu” (BL23) for 10/20/30 minutes, every other day, for 30 days	Decreased the contents of urinary microglobulin-α1, micro-albumin, transferrin and IgG, and Scr, BUN and uric acid, improved the injury of the renal tissue	Improved the kidney function and pathological changes	None
(27) Zhang et al.	Preclinical study	Male SD rats	36	DN	Needling at “Zhongwan” (CV12), “Quchi” (LI11), “Hegu” (LI4), “Zusanli” (ST36), “Yinlingquan” (SP9), “Xuehai” (SP10), “Diji” (SP8), “Sanyinjiao” (SP6), “Fenglong” (S40), and “Taichong” (LR3) for 30minutes, once daily, for 4, 8, or 12 weeks	Improved 24h-UP, BUN, TC, and triglycerides levels, the density of slit diaphragms. Promoted the renal expression of nephrin, CD2AP, and podocalyxin and decreased the expression of desmin	Improved the kidney function, and prevent the progression of DN	Ameliorated podocyte lesions
(29) Zhao et al.	Preclinical study	Male SD rats	40	Aging	Acupuncture at “Guanyuan” (CV4) and “Zusanli” (ST36) for 30 minutes, once daily, for 28 days	Reduced the contents of H_2_O_2_ and MDA in kidney tissue and kidney cell apoptosis rate	Delayed aging	Regulate peroxidation and apoptosis
(30) Cao et al.	RCT	Human	38	CRF, Anemia	Injected rHuEpo subcutaneously at “Shenshu” (BL23) and “Zusanli” (ST36), 3 times a week, for 2 months	Decreased the values of CRP, IL-6, TNF-α, Scr and BUN, increased Hb and SF levels	Improved EPO resistance and enhanced EPO efficacy, improved renal function and anemia	Alleviate micro-inflammatory state of the body
(31) Karjalian et al.	RCT	Human	90	Uremic pruritus	Applied symmetrical pressure on “Sanyinjiao” (SP6), “Xuehai” (SP10), “Zusanli” (ST36) and “Quchi” (LI11), for one minute, followed by three intermittent pressures on each point	Reduced the severity of pruritus and the levels of serum phosphorus and parathyroid hormone	Improve the severity of pruritus	None
(32) Mohammadi et al.	RCT	Human	60	RLS	NIR light was applied to “Zusanli” (ST36), “Sanyinjiao” (SP6), “Yanglingquan” (GB34) and “Chengshan” (BL57), for 2 minutes, 3 times a week, for 4 weeks	Decreased the mean RLS scores during the intervention sessions	Attenuated the symptoms of RLS in hemodialysis patients	None
(39) Wang et al. (2022)	Preclinical study	Male Wistar rats	60	DN	EA at “Guanyuan” (RN4), “Zusanli” (ST36), “Zhongwan” (RN12) and “Fenglong” (ST40) acupoints, for 15 minutes every other day, for 8 weeks	Increased the levels of body mass, SOD activity, and FoxO1 and PGC-1α expression, decreased the contents of blood glucose, Scr, BUN, ALB, MDA and ROS, and reduced pathological damage	Improved renal function, reduced the oxidative stress response and protected the kidneys	Rise the levels of forkhead transcription factor O1 and peroxisome proliferators-γ Coactivator-1α in rat mesangial cells
(40) Gao et al.	Preclinical study	Male SD rats	40	DN, Contrast-induced nephropathy	Needling/MO/needling & MO at “Sanyinjiao” (SP6), “Shenshu” (BL23) and “Pishu” (BL20), for 5/3/5&3 minutes, once daily, for 7 days	Needling & MO treatment down-regulated BUN and Scr levels, and Fas and FasL mRNA and protein expression levels, and up-regulated renal MDA, NOS, SOD and T-AOC activity	Reduced the oxidative stress and renal injury. Needling and MO has a synergistic effect	Down-regulate the expression of renal Fas and FasL genes and proteins
(41) Wang et al.	RCT	Human	120	DN	EA at “Zhongwan” (CV12), “Fenglong” (ST40), “Xuehai” (SP10) and “Taichong” (LR3), “Guanyuan” (CV4) and “Zusanli” (ST36), for 30 minutes, for 5 times a week, for 8 weeks	Decreased the levels of UAER, Scr, BUN, CysC, ηbL, ηbM, ηbH, ηp and FIB, increased serum eNOS and NO levels	Improved renal function and reduced microcirculation disorders	Up-regulate the levels of serum eNOS and NO
(42) Huang et al.	Preclinical study	Male Wistar rats	40	DN	EA at “Shenshu” (BL23) and “Zusanli” (ST36) for 20 min, 5 times a week, for 6 consecutive weeks	Decreased the levels of 24h-UP, FBG, BUN and p62, up-regulated the expression levels of LC3II, Beclin-1 and Nephrin proteins and ratio of LC3II/I. No significant change was found in the level of Scr. Improved the number of autophagosomes or autophagobubbles in podocytes	Alleviated kidney damage and improved facilitating autophagy	Improve facilitating autophagy
(43) Zhang et al.	Preclinical study	Mice	36	DN	EA at “Shenshu” (BL23), “Zusanli” (ST36) and “Sanyinjiao” (SP6), etc. acupoints for 20 minutes, once daily, for 3 weeks	Decreased the serum levels of TNF-α, IL-6, IL-1β and IL-18 in DN mice, reduced the number of renal mononuclear macrophage differentiation, changed the mRNA expression of NOS2 and Arg1, and NO production levels of renal mononuclear macrophage differentiation, suppressed the protein expression of HMGB1, NLRP3 and NF-κB in renal mononuclear macrophage	Reduce DN-induced inflammation and protect renal function	Suppressed HMGB1/NLRP3/NF-κB pathway at renal mononuclear macrophage to attenuate inflammation
(44) Zhang et al.	RCT	Human	130	DN	Needling at “Quchi” (LI11), “Zhigou” (TE6), “Hegu” (LI4), “Xuehai” (SP 10), “Zusanli” (ST36), “Yinlingquan” (SP9), “Fenglong” (ST40), “Diji” (SP 8), “Sanyinjiao” (SP6), “Taichong” (LR3), “Tianshu” (ST25), “Gaohuang” (BL43), “Shenshu” (BL23), “Zhongwan” (CV12) and “Zhongji” (CV3), for 30 minutes, twice a day, for 42 days	Improved symptoms of the patients, and had benign regulative action on metabolism of blood sugar and lipids, and GFR, renal blood flow and urinary albumin level, inhibited over expression of MCP-1	Improved renal blood flow and renal function, and protected glomerulus and renal tubules, so as to delay renal lesion	None
(45) Zhang et al.	RCT	Human	130	DN	Needling at “Zhongwan” (RN12), “Quchi” (LI11), “Hegu” (LI4), “Zusanli” (ST36), “Yinlingquan” (SP9), “Sanyinjiao” (SP6), “Fenglong” (ST40), “Xuehai” (SP10), “Diji” (SP8), “Taichong” (LR3), “Baihuanshu” (BL30), “Shenshu” (BL23), “Gaohuang” (BL43) and “Zhongji” (RN3), for 30 minutes, twice a day, for 6 weeks	Improve clinical symptoms and signs, FBG, UAER, beta2-microglobulin, MCP-1, lymphocyte membrane cholesterol, MDA, 8-OHdG, SOD, CD3+, CD4+, CD8+, and CD4+/CD8+	Improved glycometabolism disturbance-induced progressive kidney injury	Restrain overexpression of MCP-1, adjust level of oxidative stress, prohibit oxidation of protein, increase protectiveness of membrane, adjust quantity and activity abnormity of T lymphocyte subgroup, leading to repairing lymphocyte damage and improving immune expression
(46) Li et al.	Preclinical study	Male Wistar rats	12	None	EA at “Taixi” (KI3) for 20 minutes, once daily, for 7 days	Increase NAD-dependent isocitrate dehydrogenase and quinone reductase expression in the kidney	“Taixi” (KI3) has a relationship with Kidney	None
(47) Chen et al.	Preclinical study	Male Wistar rats	12	None	EA at “Taixi” (KI3) for 20 minutes, once daily, for 7 days	Increase NAD-dependent isocitrate dehydrogenase and quinone reductase expression in the kidney	“Taixi” (KI3) increased energy metabolism, and has a close relationship with kidney	None
(48) Li et al.	Preclinical study	Male C57BL/6 mice	80	DN	EA at “Zusanli” (ST36) and “Shenshu” (BL23), once daily, for 7 successive days	Down-regulated the blood glucose, alleviated the renal tissue injury, and decreased the expressions of TRPC6 and Nephrin in glomerulus and renal tissue	Alleviated renal injury	Reduce renal TRPC6 and Nephrin expressions and inhibiting podocyte activation
(49) Li et al.	Preclinical study	Male C57BL/6 mice	80	DN	EA at “Zusanli” (ST36) and “Shenshu” (BL23), once daily, for 7 successive days	Down-regulated the blood glucose, alleviated the renal tissue injury, and decreased the expressions of TRPC6 and related apoptotic proteins Caspase-3, Bax and Bcl-2 in the renal tissue	Alleviated renal injury	Down-regulate the expression of TRPC6 and Caspase-3 and up-regulating the ratio of Bcl-2/Bax
(50) Song et al.	RCT	Human	152	CKD, renal hypertension	Needling at s “Jiangya” and “Shenbing” acupoints, once daily, the interval of 3 days once every 2 weeks, for 24 weeks	Lowered blood pressure, reduced UP, decreased Scr	Improved renal function and lowered blood pressure	None
(51) An et al.	Preclinical study	New Zealand white rabbits	50	glomerulonephritis	Needling at “Fengmen” (BL12) and “Shenshu” (BL23) for 30 minutes, once daily, for 8 weeks	Lowered blood pressure, parameters of renal function and improved podocyte injury, increased the protein expression of phosphorylated ERK1/2.	Lowered blood pressure and halted deteriorating renal function	Inhibit the ERK1/2 MAPK pathway to reduce renal sympathetic nerve activity
(52) Paterno et al.	Preclinical study	Male Wistar rats	56	CKD	EA at “Zusanli” (ST-36) and “Taixi” (KI-3) and MO at “Shenshu” (BL23), for 20 minutes, twice a week, for 8 weeks	EA-MO reduced proteinuria, lowered Scr and urea concentrations, reduced glomerulosclerosis and tubulointerstitial fibrosis indices, increased in serum and renal NO levels, attenuated the elevation of TBP, MAP and RSNA	Lowered blood pressure, improved renal function and alleviated pathological damage of renal tissue	Regulate renal sympathetic nerve activity and NO level
(53) Kim et al.	Preclinical study	Male golden Syrian hamsters	12	Renal hypertension	EA at “Zusanli” (ST-36) for 30 minutes, once daily, for 5 days	Reduced MAP, increased periarteriolar NO concentration, and prevented the reduction of eNOS and nNOS	Reduced blood pressure	Activation of eNOS and nNOS reduces blood pressure through the stomach meridian
(54) Oh et al.	Preclinical study	Male SD rats	50	Renal failure, Renal hypertension	EA at “Zusanli” (ST36) and “Taixi” (KI3) acupoints, for 10 minutes, once daily, for 10 days	Reduced blood pressure, albuminuria, serum BUN and Scr concentrations, attenuated the increments of glomerulosclerosis and tubulointerstitial fibrosis, increased IGF-I mRNA and protein levels in both the kidney and the serum, and decreased the expressions of oxidative stress-related substances	Reduced blood pressure and protected renal function	be related to the effects of oxidative stress on IGF-I in renal failure-induced hypertension
(55) Yang et al.	Preclinical study	Male SHR rats	40	Hypertensive nephropathy	EA at “Shenshu” (BL23), “Geshu” (BL17) or both “Shenshu” (BL23), “Geshu” (BL17) for 15 minutes, every other day, for 12 weeks	Decreased the blood pressure and the expression levels of renal TIMP-1, PAI-1 and α-SMA proteins. Improved renal pathological damage	Reduced the blood pressure and alleviated pathological damage of renal tissue	Down-regulate expression of TIMP-1, PAI-1 and α-SMA proteins
(56) Chen et al.	Preclinical study	Male SHR rats	24	Hypertensive nephropathy	EA at “Quchi” (LI11) and “Zusanli” (ST36) acupoints for 20 minutes, once daily, for 6 weeks	Decreased the blood pressure, positive depositional area of type I and III collagen and the expression of semi-quantitative analysis of TGF-β1 mRNA	Lowered the blood pressure and improved the damage of kidney morphology	Intervenes the process of RIF by reducing synthesis of kidney type I, III collagen and restraining expression of TGF-β1.
(57) Che-Yi et al.	RCT	Human	40	Uremic pruritus, ESRD	Needling at the “Quchi” (LI11) acupoint, thrice weekly, for 1 month	Lowered pruritus scores	Relieved uremic pruritus	None
(58) Akça et al.	RCT	Human	75	Uremic pruritus, ESRD	Acupressure at the “Quchi” (LI11) acupoint, thrice weekly, for 4 weeks	Reduced the levels of discomfort from uremic pruritus	Relieved uremic pruritus	None
(59) Rehman et al.	RCT	Human	58	Uremic pruritus, ESRD	Acupressure at “Yongquan” (KI1), for 6 minutes, once daily, for 8 weeks	Reduced the PSQI score and improved the mean EQ5D index score	Improved the sleep quality and quality of life	None
(60) Arab et al.	RCT	Human	108	Uremic pruritus, ESRD	Acupressure at “Shenmen” (HT7) acupoint, for 8 minutes, 3 times a week, for 4 weeks	Reduced the total PSQI score	Improved the sleep quality	None
(61) Shariati et al.	RCT	Human	48	Sleep disorder, ESRD	Acupressure at “Shenmen” (HE7), “Hegu” (Li4) and “Sanyinjiao” (SP6) acupoints, for 9 minutes, 3 times a week, for 4 weeks	Improved the scores of PSQI, subjective sleep quality, sleep latency, sleep duration, sleep efficiency, sleep disturbance, the use of sleeping medication, and daytime dysfunction	Improved the sleep quality	None
(62) Tsay et al.	RCT	Human	106	Fatigue, ESRD	Acupressure at “Yongquan” (KI1), “Zusanli” (ST36), “Yanglingquan” (GB34) and “Sanyinjiao” (SP6) acupoints, for 12 minutes, 3 times a week, for 4 weeks	Improved the results of the revised PFS, VAS of Fatigue, PSQI and the Beck Depression Inventory	Improved fatigue	None
(63) Eğlence et al.	RCT	Human	118	Fatigue, ESRD	Acupressure at “Zusanli” (ST36), “Yanglingquan” (GB34), “Sanyinjiao” (SP6) acupoints and electrically stimulate at “Yongquan” (KI1) acupoints, 3 times a week, for 1 month	Lowered the subscale and total fatigue scores for the VAS and PFS, except for the score of cognitive subscale on the PFS	Decreased fatigue	None
(64) Wang et al.	RCT	Human	109	Comprehensive symptoms, ESRD	MO at “Zusanli” (ST 36) and “Sanyinjiao” (SP 6), 2 to 3 times a week, for 12 weeks	Increased the survival quality scores of physical functioning, general health, mental health, social functioning, vitality, effects of kidney disease and cognitive function	Improved the survival quality of physical functioning, general health and vitality, which benefits the psychological condition of the patients	None
(65) Kim et al.	RCT	Human	24	Comprehensive symptoms, ESRD	Individualized acupuncture treatments were provided twice a week, for 6 consecutive weeks	Improved the results of some subscales of KDQOL-SF, including effects of kidney disease, burden of kidney disease, role-limitations physical, emotional well-being, energy/fatigue and physical functioning	Improved the survival quality of life	None
(66) Li et al.	RCT	Human	97	Comprehensive symptoms, ESRD	MO at “Zusanli” (ST 36) and “Sanyinjiao” (SP 6) acupoints, 2 to 3 times a week, for 12 weeks	Improved the symptom scores of lassitude and fatigue, short breath and aversion to talk, poor appetite, soreness and softness of waist and knees, aversion to cold, cold extremities, etc.	Improve the clinical symptoms	None
(67) Su et al.	RCT	Human	69	Comprehensive symptoms, ESRD	FIR or HP at “Qihai” (RN6), “Guanyuan” (RN4) and “Zhongji” (RN3) acupoints, for 30 minutes, 3 times a week, for 12 weeks	Improved some parameters of the HRVA. Improved the scores of the psychological domain and the environmental domain of the WHOLQOL-BREF questionnaire	Decreased both stress and fatigue levels and stimulated autonomic nervous system activity	None
(68) Sun et al.	RCT	Human	71	Comprehensive symptoms, ESRD	MO at “Zusanli” (ST 36), “Guanyuan” (CV4) and “Sanyinjiao” (SP 6) acupoints, for 6 minutes, 2 to 3 times a week, for 12 weeks	Improved some fields of the KDQOL-SF, including role- emotional, role-physical, energy, social support, work status, quality of social interaction, etc.	Improved physical strength and mood in the quality of life	None
(69) Tsay et al.	RCT	Human	106	Comprehensive symptoms, ESRD	Acupressure/TEAS at “Zusanli” (ST 36), “Guanyuan” (CV4) and “Sanyinjiao” (SP 6) acupoints, for 13 minutes, 3 times a week, for 1 month	Improved the scores of PFS, PSQI and the Beck Depression Inventory	Lowered the levels of fatigue, a better sleep quality and less depressed moods	None
(70) Bullen et al.	RCT	Human	101	ESRD	Individual needling or massage for 20 minutes, once a week, for 8 weeks	Improved PROMIS mental raw score	Improved the health-related quality of life	None

RCT, randomized controlled study; CKD, chronic kidney disease; EA, electroacupuncture; Scr, serum creatinine; GFR, glomerular filtration rate; MO, moxibustion; 24h-UP, 24-hour urine protein; TCM, traditional Chinese medicine; TC, total cholesterol; TG, triacylglycerol; ALB, albumin; SD, Sprague Dawley; FSGS, focal segmental glomerulosclerosis; α-SMA, alpha-smooth muscle actin; TGF-β, transforming growth factor-beta; ECM, extracellular matrix; CRF, chronic renal failure; TNF−α, tumor necrosis factor−α; ILK, integrin linked kinase; IL−8, interleukin-8; eNOS, endothelial nitric oxide synthase; RIF, renal interstitial fibrosis; BUN, blood urea nitrogen; DN, diabetic nephropathy; CD2AP, CD2-associated protein; H_2_O_2_, hydrogen peroxide; MDA, malondialdehyde; CRP, C-reactive protein; Hb, hemoglobin; SF, serum ferritin; EPO, erythropoietin; NIR, near-infrared; RLS, restless legs syndrome; SOD, superoxide dismutase; FoxO1, forkhead box O1; PGC-1α, peroxisome proliferator-activated receptor-γ coactivator-1α; ROS, reactive oxygen species; T-AOC, total antioxidant capacity; UAER, urine albumin excretion rate; CysC, cystatin; ηbL, whole blood low-cut viscosity; ηbM, whole blood mid-cut viscosity; ηbH, whole blood high-cut viscosity; ηp, plasma viscosity; FIB, fibrinogen; NO, nitric oxide; FBG, fasting blood glucose; LC3, microtubule-associated protein light chain 3; Arg1, arginase-1; HMGB1, high mobility group box-1; NF-κB, nuclear factor-kappaB; MCP-1, monocyte chemoattractant protein-1; 8-OHdG, 8-hydroxydeoxy guanosine; NAD, nicotinamide adenine dinucleotide; TRPC6, transient receptor potential-6 channels; ERK1/2, extracellular signal-regulated kinase 1/2; TBP, tail-cuff blood pressure; MAP, mean arterial pressure; RSNA, renal sympathetic nerve activity; nNOS, neuronal nitric oxide synthase; IGF-I, insulin-like growth factor-I; SHR, spontaneously hypertensive rats; TIMP-1, tissue inhibitor of metalloproteinase 1; PAI-1, plasminogen activator inhibitor-1; PSQI, pittsburgh sleep quality index; PFS, piper fatigue scale; VAS, visual analog scale; KDQOL-SF, kidney disease quality of life-short form; HP, heat pad; HRVA, heart rate variability analyzer; WHOLQOL-BREF, a questionnaire authenticated and approved by the World Health Organization; TEAS, transcutaneous electrical acupoint stimulation; PROMIS, patient-reported outcomes measurement information system.

## 3 Results

**Figure 1:**
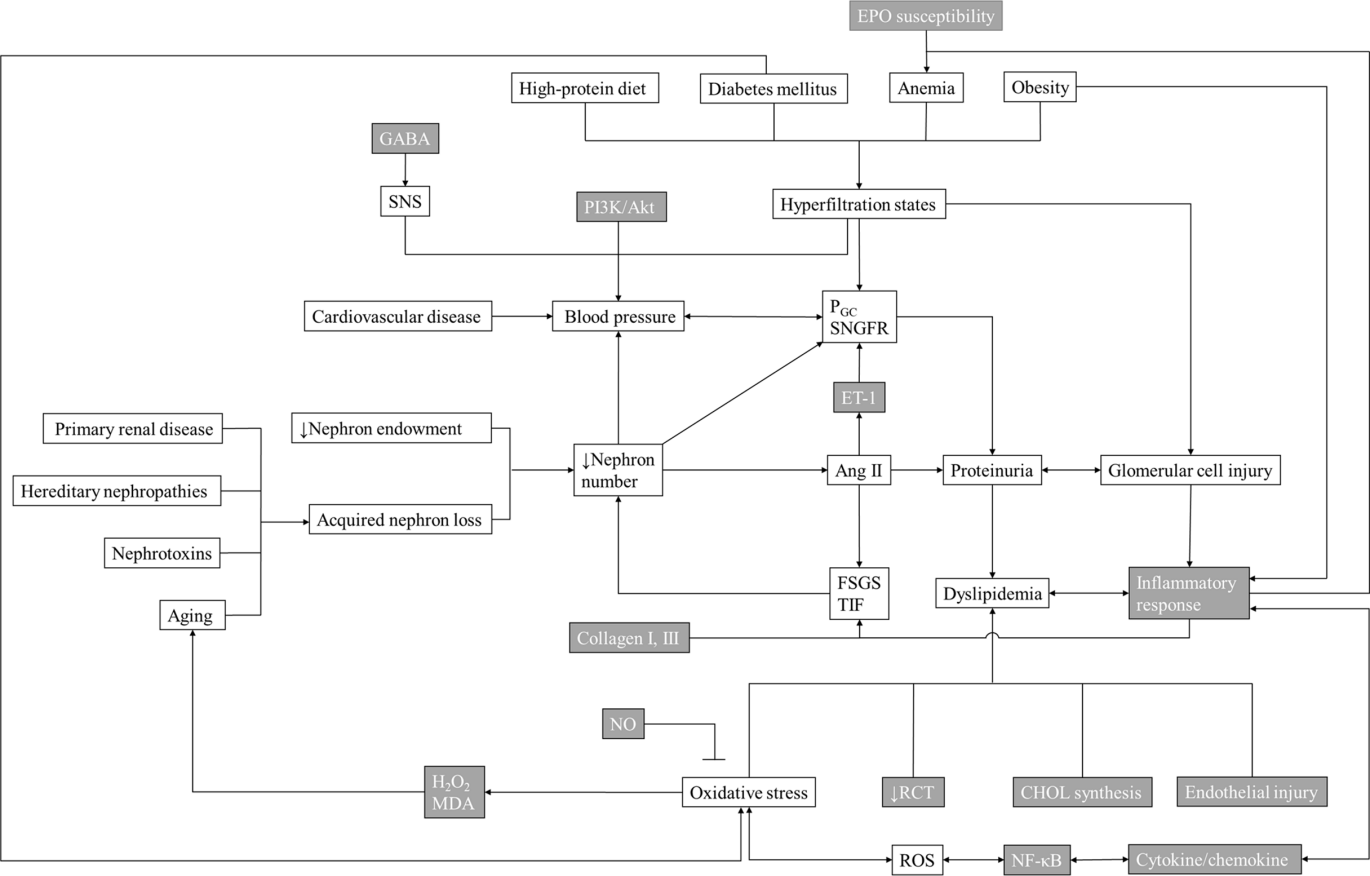
Mechanisms Underlying the Effects of Acupuncture Therapy in Chronic Kidney Disease. The gray grids are the known possible main mechanism/target/downstream product of acupuncture in treating chronic kidney disease. EPO, erythropoietin; GABA, γ-aminobutyric acid; SNS, sympathetic nervous system; PGC, proliferator γ coactivator; SNGFR, single nephron glomerular filtration rate; ET-1, endothelin-1; Ang II, angiotensin II; FSGS, focal segmental glomerulosclerosis; TIF, tubulointerstitial fibrosis; MDA, malondialdehyde; RCT, reverse cholesterol transport; CHOL, cholesterol; NF-κb, nuclear factor-kappa B; ROS, reactive oxygen species.

The 47 studies included in this review employed the use of Sprague Dawley rats, Wistar rats, Golden Syrian hamsters, New Zealand white rabbits, which were used to model ischemic nephropathy, diabetic nephropathy (DN), or hypertensive nephropathy. According to the risk factors of CKD, including hyperglycemia, hypertension, hyperlipidemia, obesity, aging, and anemia (25), we classified and summarized the potential mechanisms of action of the beneficial effects of acupuncture on the progress of CKD (**Figure 1**) and the common symptoms of ESRD.

### 3.1 Risk factors of CKD and the corresponding mechanism of acupuncture

#### 3.1.1 Hyperglycemia

DN is rapidly becoming the most common cause of ESRD worldwide (25). The pathogenesis of DN is complex and involves various mechanisms, resulting in poor therapeutic outcomes (71). It is generally accepted that the developmental mechanism of DN results from abnormal homeostasis (72). There are many critical links in the progression of DN, e.g., oxidative stress, inflammation, and podocyte structural damage. A reciprocal relationship exists between inflammation and oxidative stress (73, 74). Various Chinese meta-analyses point out that acupuncture can reduce the urine protein, serum creatinine, fasting blood glucose, postprandial blood glucose, glycosylated hemoglobin, total cholesterol, and triglycerides of patients with DN, as well as enhance the efficacy when combined with conventional drugs (26, 75, 76).

##### 3.1.1.1 Oxidative stress

The increase in reactive oxygen species (ROS) caused by blood glucose is at the core of the pathogenesis of DN. Hyperglycemia-induced oxidative stress is believed to cause both local and systemic inflammation (77).

Forkhead transcription factor O1 (FOXO1) overexpression reduced ROS in rat mesangial cells and protected mitochondrial function by activating peroxisome proliferator γ coactivator 1 α (PGC-1α) (78). A study on rats with DN showed that electroacupuncture (EA) increased the levels of FOXO1 and PGC-1α in kidney tissue, thus improving renal function (39).

Nitric oxide synthase (NOS), superoxide dismutase (SOD), and malondialdehyde (MDA) are essential indicators of oxidative stress. Reactive species may also be produced enzymatically by uncoupled NOS (79). SOD is an antioxidant that can effectively remove superoxide anions and protect cells from oxidative damage (80). MDA is a metabolite of lipid peroxidation, which can reflect the level of free radicals in tissues and lipid peroxidation caused by free radicals, and indirectly reflects cell damage (40). A study on rats with DN showed that acupuncture and moxibustion have synergistic effects on antioxidant stress, which may be related to their function in downregulating the expression of MDA and upregulating the expressions of NOS and SOD in the kidney (40). Another trial revealed that EA could improve renal function and reduce microcirculation disorders in early DN by up-regulating the levels of serum endothelial NOS and NO (41).

Oxidative stress can directly damage podocytes, mesangial cells, and endothelial cells, resulting in proteinuria and tubulointerstitial fibrosis. One study suggested that EA may effectively alleviate renal injury in rats with DN by promoting renal autophagy. The number of podocytes in rats with DN treated with EA was more than that in the untreated group, while the levels of 24-h urine protein, blood urea nitrogen, and serum creatinine were lower (42).

Nuclear factor 2 related factor 2 (Nrf2) regulates oxidative stress in the antioxidant response system by controlling the expression of more than 250 genes (72, 79). A study on rabbits with acute kidney injury revealed that EA treatment enhanced the expression of phosphorylated Akt, heme oxygenase-1 protein, Nrf2 total protein, and nuclear protein to resist oxidative stress (81).

##### 3.1.1.2 Inflammation

Continuous inflammation of the circulatory system and renal tissue is the fundamental pathological basis for the development of DN (82). Inflammatory factors such as interleukin-6 (IL-6), tumor necrosis factor-α (TNF-α), transforming growth factor-β, IL-1, and IL-18 are elevated in the blood and have been related to the occurrence and progression of DN (83, 84). Several studies have shown that acupuncture can improve insulin resistance by reducing serum IL-6, IL-8, and IL-1β levels, which might help protect islet B cell function (43, 85).

Increasing evidence has shown the central role of Janus kinase (JAK)-signal transducer and activator of transcription (STAT) pathway (71) in DN pathogenesis. JAK and STAT subtypes expressed on the renal tubulointerstitial increase along with the development of DN and negatively correlated with the estimated GFR. Nuclear factor-kappa B (NF-κB) is a key transcription factor in the inflammatory process of DN and is activated by the JAK-STAT pathway. NF-κB regulates inflammatory cytokines and chemokines, e.g., monocyte chemoattractant protein-1 (MCP-1) and cell adhesion proteins, leading to kidney damage. A study on diabetic mice revealed that acupuncture could suppress the inflammatory response of DN through the NF-κB-related pathway (43). Zhang et al. designed a series of multicenter, randomized, and blinded studies, showing that the needling method of harmonizing the spleen and stomach on patients with early DN might inhibit the NF-κB-related pathway by inhibiting the expression of MCP-1, which can improve renal blood flow and GFR, decrease urinary albumin secretion, protect the glomerulus and renal tubules, thus reducing the inflammatory levels and delaying the progress of DN (44, 45).

##### 3.1.1.3 Energy metabolism

The kidney requires a large number of mitochondria to provide the energy to remove waste from the blood and regulate fluid and electrolyte balance. Mitochondrial dysfunction leads to a decrease in ATP production, alterations in cellular functions and structure, and the loss of renal function (86, 87). Figueiredo et al. (88) found that in non-exercised hyperglycemic rats, under the same dose of anesthesia (ketamine, 90 mg/kg body weight), the lactic acid concentration and blood glucose level of the experimental group treated with EA decreased significantly, indicating that acupuncture might reduce blood glucose by enhancing aerobic metabolism and increasing ATP output.

NAD-dependent isocitrate dehydrogenase is present in the mitochondria. It is a momentous rate-limiting enzyme of the tricarboxylic acid cycle (TCA) and plays a crucial role in energy production and anabolism. As the most important source of adenosine triphosphate (ATP), TCA is closely related to the occurrence of nephropathy. Two other studies reached similar conclusions; after needling the “Taixi” (KI 3) point, increased expression of NAD-dependent isocitrate dehydrogenase and quinone reductase was observed in rat kidney tissue, suggesting that targeted acupuncture improves energy metabolism (46, 47).

Metabonomics has also been applied to study EA’s effects on renal metabolism. Alanine is a characteristic metabolite in the kidney, an important energy source for human beings, and is involved in lymphocyte regeneration, thus maintaining immune homeostasis (89). Threonine participates in energy metabolism and promotes the cellular immune system’s defense function (89). Research suggested that the levels of the two metabolites in the kidneys of mice with premature ovarian failure were elevated, and the levels were down-regulated after electroacupuncture stimulation of “Sanyinjiao” (SP6) and “Guanyuan” (CV4), close to the level of healthy mice (90). Another study revealed that the effect of EA on the abnormal increase of metabolites might suggest that it can regulate the disordered amino acid metabolism, thereby improving energy metabolism and regulating the kidney’s immune function (91).

##### 3.1.1.4 Maintaining the podocyte structure

Podocytes are important functional cells in the glomerulus that cannot regenerate when they suffer from injury. Their damage and apoptosis could result in the destruction of the glomerular filtration membrane and induce DN (92). Podocalyxin is one of the main structures responsible for the negative charge on the glomerular membrane (93). CD2-associated protein (CD2AP) is a transmembrane protein that interacts with nephrin to maintain cytoskeleton and slit diaphragm function. Damage to CD2AP leads to the destruction of the podocyte skeleton and marked proteinuria (94). Desmin also maintains the mechanical stability of podocytes to enable morphological changes on the tensile glomerular capillary wall (95). Zhang et al. found that acupuncture partly prevented DN rats from podocyte foot process effacement—which exhibited fusion, complete destruction, or disappearance—and thick glomerular basement membrane. It also upregulates nephrin expression, CD2AP, and podocalyxin but downregulates desmin, thus protecting and maintaining podocytes’ physical and chemical structure (27).

The transient receptor potential-6 channel (TRPC6) is an integral player in the calcium processing in podocytes and in the maintenance of their cellular structure (96, 97). Möller et al. reported that overexpression of TRPC6 in healthy mice leads to the restructuring of the podocyte actin cytoskeleton and alterations in calcium flux, which causes proteinuria (97). Hyperglycemia and elevation in angiotensin II (Ang II) levels are sufficient to cause overexpression of TRPC6, resulting in increased calcium influx and eventual podocyte dysfunction and death (98). Li et al. proved that EA preconditioning could alleviate renal injury in hyperglycemic mice by reducing renal TRPC6 and nephrin expression and inhibiting podocyte activation (48, 49).

#### 3.1.2 Hypertension

Hypertension is considered a result of renal damage and a significant contributor to the progression of CKD (25). Approximately 80–90% of patients with CKD have renal hypertension, which accelerates renal dysfunction (99, 100). Therefore, controlling blood pressure is critical in preventing progressive deterioration of renal function. Renal hypertension is difficult to cure and usually requires the combined use of several antihypertensive drugs with possible non-compliance. The pathological mechanism of renal hypertension is complex, including activation of the sympathetic nervous system (SNS) and renin-angiotensin-aldosterone system (RAAS), oxidative stress, increased endothelin-1 (ET-1), and inflammation. Acupuncture has been shown to have certain clinical effects on renal hypertension (50). A network meta-analysis on acupuncture therapy for essential hypertension, which included 31 trials with 2,649 patients, revealed that acupuncture might have similar effects as common medication. However, the quality of this evidence is not high (101).

##### 3.1.2.1 Angiotensin II type 1 receptor-ET-1-endothelin-1 type A receptor pathway

ET-1 is a crucial molecule that regulates renal hypertension, and its release is induced by the combination of Ang II and angiotensin II type 1 receptor (AT1R). ET-1 combines with endothelin-1 type A receptor (ETAR), which causes marked renal vasoconstriction (102). A previous study revealed that Ang II and ET-1 receptor blockers could reduce blood pressure in animal models and patients (103). Acupuncture reduces blood pressure by lowering the ET-1 level (104, 105). Additionally, long-term EA blocks the AT1R-ET-1-ETAR pathway by inhibiting the expression of AT1R and ETAR (106). Therefore, it is believed that the AT1R-ET-1-ETAR pathway may be a target for acupuncture treatment of renal hypertension.

##### 3.1.2.2 Renin-angiotensin-aldosterone system

The RAAS is a vital blood pressure regulation system that maintains the homeostasis of water and electrolytes in the internal environment. There are two main pathways. 1) The angiotensin-converting enzyme/Ang II (ACE/Ang II) pathway constricts blood vessels and promotes tissue proliferation and remodeling (107). 2) The ACE2/Ang-(1-7) pathway has the opposite effects (108). Moreover, Liu et al. found that acupuncture and moxibustion showed good antihypertensive effects by reducing the content of Ang II and atomic layer deposition in the plasma of hypertensive rats (109).

##### 3.1.2.3 SNS

The development of hypertension partly depends on the increased sympathetic outflow and impaired baroreflex function. The nucleus tractus solitarii (NTS) is the main integration center regulating the autonomic reflex and sympathetic outflow. In the NTS, inhibition of γ-aminobutyric acid (GABA) is essential for pressure reflection signal processing. Evidence shows that an increase in GABA inhibition leads to hypertension (110–112). Therefore, neuronal activity in the NTS is a significant target for acupuncture to regulate the sympathetic excitatory reflex function. A study revealed that EA could reduce sympathetic activity and significantly inhibit the sympathetic excitatory reflex in rats, which may be achieved by regulating functional GABA (113). Moreover, acupuncture reduced local renal sympathetic nerve activity by inhibiting the extracellular regulated protein kinase ½-MAPK pathway to lower blood pressure (51). Another study also verified the relationship between acupuncture and renal sympathetic activity (52).

##### 3.1.2.4 Oxidative stress

Renal hypertension induced by ischemic nephropathy is affected by oxidative stress mechanisms involving molecules such as NOS and heme oxygenase (HO-1/2) (114–116). In one study, EA was shown to prevent the reduction of endothelial NOS and nitric NOS levels associated with hypertension (53).

Inducible NOS (iNOS) and HO-1/2 expression is involved in the secretion of insulin-like growth factor-I (IGF-I) in MCF-7 cells (117). IGF-I has been proved to be related to proliferation, differentiation, survival, apoptosis, and cell protection related to oxidative stress (118). Another study showed that EA can reduce the levels of iNOS and HO-1/2 and upregulate IGF-1 levels, thus reducing glomerulosclerosis and renal interstitial fibrosis as well as blood pressure in rats with renal failure (54). Additionally, the possibility of acupuncture being able to directly affect the process of renal fibrosis in hypertensive rats has also been suggested (55, 56).

#### 3.1.3 Hyperlipidemia/obesity

Recent studies have shown that hyperlipidemia and obesity are two adverse factors associated with the progression of CKD *via* different mechanisms. However, obese patients are typically at higher risk of hyperlipidemia (119). Obesity is associated with high glomerular filtration and other glomerular hemodynamic alterations, which may aggravate CKD progression (120, 121). Adipocytes produce various hormones and pro-inflammatory molecules, which may lead to progressive renal damage (122). According to the lipid nephrotoxicity hypothesis, hyperlipidemia can lead to inflammation, oxidative stress, and endogenous electrical stress (123).

A study on 1528 obese patients with hyperlipidemia treated with acupuncture suggested that acupuncture had dual effects on obesity and hyperlipidemia. The patients not only effectively lost weight (the total effective rate of the mild obesity group was 98.9%), but they also reduced their levels of total cholesterol, triglycerides, and low-density lipoprotein and improved their high-density lipoprotein (HDL) level (28). Some scholars believe that acupuncture combined with moxibustion reduces the adverse effects of hyperlipidemia and obesity better than acupuncture alone (124). Another study found that different acupoint combinations had different effects on reducing blood lipid levels. The “Quchi” (Li 11), “Zhongwan” (CV 12), and “Fenglong” (ST 40) points had a superior performance on blood lipid metabolism (125).

##### 3.1.3.1 Anti-oxidative stress

Nitric oxide (NO) is an endothelium-derived messenger molecule that alleviates oxidative stress. Several studies revealed that EA and moxibustion increase the level of NO to resist oxidative stress and that the effect of moxibustion is regulated by temperature (126–130). Transient receptor potential vanilloid subfamily 1 (TRPV1) is an essential molecular regulator that provides moxibustion with temperature dependence of its hypolipemic properties. There is a relationship between the cholesterol-lowering effect of moxibustion and the activation of TRPV1 (131).

##### 3.1.3.2 Reverse transport mechanism

Reverse cholesterol transport (RCT), which is partly mediated by ATP-binding cassette transporter A1 (ABCA1), is a significant physiological link that delays hyperlipidemia progression. ABCA1 regulates intracellular RCT and HDL production, thereby controlling lipid metabolism. As transcription factors, activated peroxisome proliferator-activated receptor (PPAR)-α and liver X receptor α (LXRα) enhance ABCA1 transcription activity (132, 133). Zou et al. believed that moxibustion upregulated PPARγ and scavenger receptor B1 (SR-B1) protein and gene expression in the liver to promote cholesterol reversal (134). HDL binds to SR-B1 and transports cholesterol to the liver for selective metabolism. Another research study found that EA stimulation of the “Fenglong” (ST 40) point contributed to increased expressions of ABCA1, PPARα, LXRα, and retinoid X receptor α messenger RNA, thus contributing to RCT, and somehow had a therapeutic effect on hyperlipidemia (135, 136).

ABCA1 dysfunction leads to excessive cholesterol ester accumulation as lipid droplets in macrophages, thereby contributing to foam cell formation (137). EA at the “Fenglong” (ST 40) point can prevent macrophage transformation into foam cells and increase cholesterol outflow rate in macrophages, thus preventing and reversing foam cell formation (138).

##### 3.1.3.3 Lipids synthesis reduction

Sterol regulatory element binding protein-1C (SREBP-1C) is a transcription factor involved in the transcriptional regulation of the fatty acid synthase (*FAS*) gene that controls the synthesis of lipids from glucose in the liver (139). Recent research indicated that *FAS* could catalyze the *de novo* synthesis of fatty acids and impact liver physiology through signaling and energy storage (140). It is believed that acupuncture downregulates SREBP-1C and *FAS* to control the expression of key enzymes regulating cholesterol synthesis in the liver to prevent hyperlipidemia (141).

##### 3.1.3.4 Inflammation reduction

CKD is an inflammatory state that results in glomerular and tubular lesions and adversely affects lipid balance (142, 143). Several inflammatory markers have been associated with lipid levels (144). Some studies revealed that acupuncture could reduce intercellular cell adhesion molecule-1, MCP-1, TNF-α, IL-6, and IL-1γ, slowing the inflammatory process (145–147).

Adiponectin (ADPN) is the only adipocyte-specific protein negatively associated with obesity. It has anti-diabetic, antiatherosclerotic, anti-inflammatory, and antiangiogenic properties. Hand acupuncture and EA intervention positively affect hyperlipidemia by reducing blood fat content and upregulating serum HDL-C and ADPN levels in hyperlipidemic rats (148).

#### 3.1.4 Aging

A longitudinal study among individuals without nephropathy found that the GFR decreases with age, indicating that nephron loss might be part of normal aging (149). Other studies have shown that proteinuria, CKD, and ESRD incidence rates increase with age (149–151). Acupuncture can delay the aging of the kidney tissue by suppressing oxidative stress and reducing apoptosis. An experiment suggested that the apoptosis rate of renal cells and the levels of hydrogen peroxide and malondialdehyde decreased after acupuncture in adult rats (29).

#### 3.1.5 Anemia

Renal anemia is one of the most common complications of CKD and affects the quality of life and survival time of patients with CKD (152). In a study of 131 patients with CKD, elevated hemoglobin levels were independently associated with reduced mortality (153). Renal anemia is usually caused by the hyposecretion of erythropoietin (EPO). EPO is a protein hormone synthesized by proximal convoluted tubular cells, essential for erythrocytes’ growth. Medical treatments sometimes show poor efficacy, including recombinant human erythropoietin and polysaccharide iron complexes (154). Therefore, there is an urgent need for supplemental therapies to improve the curative effect. One study found that acupoint injection could reduce the level of C-reactive protein, improve the micro-inflammatory state, and help reduce EPO dosage, which is better than the traditional injection method (30). Acupoint injection reduces costs and meets the requirements of patients’ health and the economy.

### 3.2 Control of ESRD-related complications

CKD and ESRD have attracted attention worldwide, and the number of patients on hemodialysis (HD) has increased dramatically. Patients on HD often have many painful complications, such as UP, RLS, insomnia, fatigue, sleep disorders, and hypotension. Acupuncture can alleviate these problems. A systematic review of randomized controlled trials showed that acupuncture had demonstrated efficacy in alleviating sleep disturbance, fatigue, and UP symptoms among patients with CKD (36).

#### 3.2.1 Pain

Pain can be one of the most debilitating symptoms of CKD (155). Patients suffer several types of pain, including peripheral neuropathic pain, joint pain, autosomal dominant polycystic kidney disease (ADPKD)-related pain, and pain caused by renal biopsy. A multicenter, cross-sectional study evaluated the impact of pain on the quality of life of patients with ESRD on HD, with the results suggesting that pain significantly impacted their life quality (156). Pain management in patients with CKD is challenging. Non-opioid analgesia using acetaminophen, topical analgesics, and gabapentinoids is preferred, but cannot relieve pain to a great extent (155). Furthermore, the long-term use of non-steroidal, anti-inflammatory drugs poses a risk of liver and further kidney damage. A study on over 400,000 patients with ESRD showed that an opioid prescription was accepted by over half of them (157), even though opioid use is associated with an increased risk of altered mental status, falls, fractures, hospitalizations, and mortality, in a dose-dependent manner (158, 159).

Nevertheless, opioids play a central role in the analgesic mechanism, desensitizing peripheral nociceptors, reducing pro-inflammatory cytokines, and activating the descending inhibitory system (160). A previous study showed that high- and low EA frequencies could relieve heat, mechanical, and spontaneous pain by regulating μ and δ opioid receptors (161). This shows that acupuncture has the prospect of reducing or even replacing opioid use.

Besides, the role of acupuncture in the treatment of chronic lower back pain has been well proven and addressed in the Clinical Practice Guideline of the American College of Physicians (162). It is also effective against chronic lower back pain caused by polycystic kidney disease (163). Unfortunately, at present, there is a lack of large-scale studies on verifying the efficacy of acupuncture and moxibustion on lower back pain in other types of CKD.

#### 3.2.2 Uremic pruritus

Chronic pruritus associated with ESRD is one of the most important causes of systemic pruritus (164). UP is an unpleasant and painful condition causing the desire to scratch, invalidating the skin’s protective barrier, and affecting patients’ health-related quality of life (HR-QOL) (31). A large multicenter study of 18801 patients on HD showed that 42% of them had UP (165). The pathogenesis of UP may involve inflammatory states, such as increased levels of pro-inflammatory cytokines (IL-6, IL-2, and TNF-α), immune changes, and neuropathy. Current drug treatment regimens (e.g., antihistamines, bupropion, and tacrolimus) often lead to many side-effects, including sleepiness, nausea, vomiting, and epilepsy. Patients are unlikely to extract many benefits, and the symptoms usually recur after drug discontinuation (164). Acupuncture, an economical and safe complementary therapy, has obvious advantages in treating pruritus, with one meta-analysis even suggesting the potential of acupuncture to treat UP (166). Studies have shown that one of the mechanisms of acupuncture in treating chronic pruritus is regulating inflammatory cytokines, including reducing IL-4 and IL-2 levels in serum, enhancing the anti-inflammatory cytokine IL-10, and inhibiting the level of the inflammatory cytokine TNF-α (the acupoints are all “Quchi” (LI11), “Hegu” (LI4), “Xuehai” (SP10), and “Yinlingquan” (SP9)) (167–169). Another animal study found that EA increased the serum levels of interferon-γ of mice with atopic dermatitis, with no significant change in IL-4 levels (the acupoints are “Quchi” (LI11) and “Neiguan” (PC6)) (170). This finding suggests that different acupoints lead to different effects.

Karjalian et al. conducted a randomized, double-blind, pre- and post-control clinical trial among 90 patients on HD and found that acupoint pressing could effectively reduce pruritus in patients on HD (31). Four other randomized controlled trials showed that acupuncture, acupoint pressing, transcutaneous acupoint electrical stimulation, and auricular finger pressing could reduce UP symptoms (57–59, 171). Yi et al. believed this effect could continue after the treatment course (57).

#### 3.2.3 Restless leg syndrome

RLS is a common chronic sensorimotor disorder characterized by a strong demand to move the legs during rest and bedtime. The development of this disease in patients undergoing HD is progressive (32). The prevalence among European and American adults varies from 7% to 10% (172, 173). Further, the prevalence of RLS has been reported to increase with age (174). RLS affects sleep quality and leads to dysfunction of emotion, cognition, energy, and other daily activities. RLS is associated with an increased risk of cardiovascular disease, osteoporosis, musculoskeletal pain, and mortality (175–177). A single-center, single-blinded, randomized controlled study attempted to treat 60 HD patients with RLS by irradiating lower limb acupoints with near-infrared light, but the symptoms of RLS recurred after irradiation was stopped (32). The mechanism was unknown, and the effects might have been related to the selection of acupoints, treatment cycle, and treatment mode, which require further study.

#### 3.2.4 Sleep disorders

Sleep disorders in patients on HD can lead to psychosocial function and interpersonal relationship disorders and reduce their HR-QOL (60). More than 85% of patients on HD experience serious sleep problems (178). Zahra et al. and Shariati et al. found that acupoint pressing positively affects sleep quality in patients on HD, but the specific mechanism is still unknown (60, 61).

Acupuncture has been proved to have a significant effect on sleep disorders. Various meta-analyses, which included thousands of patients each, suggested that acupuncture could improve the sleep quality of patients with primary insomnia and patients with insomnia-related primary diseases and conditions (stroke, ESRD, perimenopause, pregnancy, and mental illness) of all ages, with few and mild adverse reactions (33, 179–182). However, because most of the included studies were heterogeneous and the sample size of each experiment was small, and there are few studies related to ESRD, it is still necessary to design randomized controlled trials with larger sample sizes to prove the essential of acupuncture to treat sleep disorders in patients on HD.

#### 3.2.5 HR-QOL

HR-QOL is a measure of the value assigned to duration of life as modified by impairments, functional states, perceptions and opportunities, as influenced by disease, injury, treatment and policy (183). Due to the progress of ESRD, the lifestyle restrictions and changes imputed to HD usually lead to fatigue, depression, anxiety, which have a profound impact on HR-QOL. The mental health of elderly patients is an especially serious problem (184). Mid- and long-term fatigue may even increase the risk of cardiovascular events and is associated with higher mortality (185). The clinical trials of this subsection were evaluated through the following questionnaires: Pittsburgh Sleep Quality Index, Piper Fatigue Scale; Visual Analog Scale, Kidney Disease Quality of Life-Short Form, a questionnaire authenticated and approved by the World Health Organization and Patient-Reported Outcomes Measurement Information System. Two studies showed that acupoint pressing could improve the fatigue state of HD patients (62, 63), and several randomized controlled studies found that moxibustion, acupressure, transcutaneous electrical acupoint stimulation, and acupuncture had positive impacts on aspects such as physical functions, general health, and vitality (64–69). However, in 2018, a study on living conditions of 101 HD patients who received short-term acupuncture/massage suggested that although the original Patient-Reported Outcomes Measurement Information System psychological scores of patients improved, the improvement was not significant, and might have been related to the sample capacity and treatment duration (70).

## 4 Discussion

CKD is a significant public health problem worldwide and is characterized by a high incidence rate and complex pathogenesis. The clinical treatment scheme needs to be improved urgently, as currently the main clinical therapeutic strategy is to delay the progress of CKD, which mostly relies on medication. According to Kidney Disease Improving Global Outcome (KDIGO) guidelines, dealing with the complications of CKD (e.g. renal hypertension, hyperlipidemia, proteinuria) is essential, and the use of angiotensin-converting enzyme inhibitors, angiotensin receptor antagonists, and statins is common (5). Different medication schemes, including glucocorticoids, cytotoxic drugs, immunosuppressants, and biological agents are used to treat patients with CKD after assessing their renal pathologies and personal conditions (5). Moreover, if the kidney damage is secondary to other basic diseases, such as diabetes, it is necessary to control the primary disease (186). However, an ideal treatment plan has not yet been found, and the drugs used are usually accompanied by several side-effects, including digestive tract reaction, obesity, liver and kidney damage, bone marrow suppression, and reproductive damage; furthermore, the therapeutic effects may take weeks to months to manifest, which often leads to intolerance (187). About 25% of patients with CKD will eventually progress to ESRD within 20 years of diagnosis and need renal replacement therapy (4).

The evidence of this review reveals several beneficial effects of acupuncture on CKD and ESRD-related symptoms, and a summary of studies on acupuncture therapy in chronic renal injury, renal physiological function and ESRD-related symptoms is presented in **Table 1**. It is believed that acupuncture may have a significant impact on the risk nodes of the progress of CKD through multiple pathways, so as to improve the prognosis of patients with CKD. This is mainly realized through the following mechanisms: 1. reduced inflammatory reactions and protection of podocytes, mesangial cells, and endothelial cells from antioxidant stress; 2. delaying glomerular and tubular lesions by downregulating inflammatory factors to regulate relevant signal pathways, including NF-κB-related pathways; 3. reducing podocyte apoptosis and protecting the glomerular filtration membrane by reducing renal TRPC6 levels and maintaining podocyte structural proteins; 4. improving glomerular hemodynamics through blood pressure regulation systems (SNS, RAAS, etc.); 5. improving energy metabolism to regulate renal immune function *via* regulating enzymes involved in aerobic metabolism in mitochondria. The major mechanisms by which acupuncture can relieve ESRD-related symptoms are: 1. activating the descent inhibition system to relieve pain by regulating the release of bioactive chemicals, especially opioids; 2. regulating inflammatory cytokines to relieve chronic pruritus.

This review fully illustrates the advantages of acupuncture in treating CKD. First, acupuncture can act in cooperation with drug therapy to improve its the curative effects (18). Second, the potential mechanisms by which acupuncture may help in treating CKD are diverse; compared with single target therapy, acupuncture improves CKD prognosis through various pathways. Third, acupuncture is a simple supplementary therapy with mild side-effects, which prevent patients from taking additional drugs, especially opioids (161), to improve patient compliance. Fourth, the schedule of acupuncture treatment is flexible and varied (**Table 1**). Fifth, acupuncture has a wide range of applications with few contraindications and is suitable for the elderly and children (35, 188). Sixth, the cost of acupuncture in China is low, which most patients can afford regardless of economic status.

Furthermore, we focused on other methods that have the potential to help improve the quality of life or relieve symptoms of CKD, including taking TCM prescriptions and Chinese patent medicine, including *Tripterygium Wilfordii Hook. f.* and *Artemisinin*, which can effectively reduce albuminuria and protect kidney tissue (189, 190). However, the compositions of traditional Chinese herbs are extremely complex; moreover, patients with advanced CKD may suffer from electrolyte disorders due to metabolic issues. Therefore, more detailed studies and monitoring is required to ensure the safety of patients. The KDIGO guidelines suggest that proper exercise and dietary management, including low sodium, high-quality protein, and low-fat diets, can help improve patients’ quality of life; acupuncture should also be recommended as an effective physical therapy (5).

There are some obvious limitations of this literature review. First, because of the involvement of various acupuncture points, complex physiological aspects, and differing patient characteristics and disease processes, acupuncture-based treatment of CKD is highly personalized, and the specific therapeutic mechanism and indications needs further study. Second, there is still a lack of large-scale, double-blind, multicenter, large sample size, randomized studies to verify the observed effects and provide a high-quality theoretical basis for them. Third, the mechanisms of acupuncture in improving ESRD-related symptoms, especially RLS and sleep disorder, cannot be clarified for now. Finally, the review could only cover studies that focused on therapeutic mechanisms that were not specific to renal pathology, on which there are very few studies. Some literatures mentioned renal interstitial fibrosis without specific pathological diagnosis. Only one clinical trial revealed the effectiveness of moxibustion on membranous nephropathy. Two animal studies mentioned focal segmental glomerulosclerosis (FSGS), one research revealed that moxibustion delay the progress of FSGS *via* alleviating podocyte injury, the other one did not mention the underlying mechanism.

## 5 Conclusions

This review suggests that acupuncture can be beneficial for CKD through several mechanisms, including oxidative stress inhibition, reducing inflammatory effects, improving hemodynamics, maintaining podocyte structure, and increasing energy metabolism. In general, acupuncture has the potential to become a new, simple, safe, and inexpensive treatment modality that can be used to treat CKD, slow the progress of renal dysfunction, and improve patient symptoms. However, the review only covers non-specific therapeutic mechanisms, lacking content related to renal pathology due to a lack of studies on this topic. Moreover, it is unclear whether acupuncture can improve CKD with different pathologies, and rigorous clinical and mechanistic studies are required to design future protocols for the use of acupuncture in such cases. This could prove conducive to understanding the potential mechanisms involved in different renal pathological diagnosis as well as the impact that acupuncture may have on them.

## Author contributions

All authors contributed to one or more of the following aspects of the manuscript: conception, acquisition of data, drafting, and revising the article. WZ and XL researched data and wrote the manuscript. XW and HM reviewed the manuscript. All authors contributed to the article and approved the submitted version.

## Conflict of interest

The authors declare that the research was conducted in the absence of any commercial or financial relationships that could be construed as a potential conflict of interest.

## Publisher’s note

All claims expressed in this article are solely those of the authors and do not necessarily represent those of their affiliated organizations, or those of the publisher, the editors and the reviewers. Any product that may be evaluated in this article, or claim that may be made by its manufacturer, is not guaranteed or endorsed by the publisher.
